# Effects of Smart Drugs on Cholinergic System and Non-Neuronal Acetylcholine in the Mouse Hippocampus: Histopathological Approach

**DOI:** 10.3390/jcm11123310

**Published:** 2022-06-09

**Authors:** Ryusei Satoh, Kiyoharu Kawakami, Kazuhiko Nakadate

**Affiliations:** Department of Basic Science, Educational and Research Center for Pharmacy, Meiji Pharmaceutical University, 2-522-1 Noshio, Kiyose 204-8588, Tokyo, Japan; rsdelight58@gmail.com (R.S.); urayuraharuki@yahoo.co.jp (K.K.)

**Keywords:** smart drugs, nootropic drug, cognitive enhancer, hippocampus, acetylcholine, choline acetyltransferase, muscarinic receptors, perivascular edema, learning, immunoelectron microscopy

## Abstract

In recent years, people in the United States and other countries have been using smart drugs, called nootropic or cognitive enhancers, to improve concentration and memory learning skills. However, these drugs were originally prescribed for attention-deficit hyperactivity disorder and dementia, and their efficacy in healthy people has not yet been established. We focused on acetylcholine in the hippocampus, which is responsible for memory learning, and elucidate the long-term effects of smart drugs on the neural circuits. Smart drugs were administered orally in normal young mice for seven weeks. The hippocampus was sectioned and compared histologically by hematoxylin and eosin (HE) staining, immunohistochemistry for acetylcholine, and immunoelectron microscopy. There were no significant changes in acetylcholinesterase staining. However, in HE, we found perivascular edema, and choline acetyltransferase staining showed increased staining throughout the hippocampus and new signal induction in the perivascular area in the CA3, especially in the aniracetam and α-glyceryl phosphoryl choline group. Additionally, new muscarinic acetylcholine receptor signals were observed in the CA1 due to smart drug intake, suggesting that vasodilation might cause neuronal activation by increasing the influx of nutrients and oxygen. Moreover, these results suggest a possible new mechanism of acetylcholine-mediated neural circuit activation by smart drug intake.

## 1. Introduction

For a long time, human beings have devised ways to improve their health and ability, such as the selection of foods rich in beneficial nutrients and ingestion of healthy foods and supplements. Improvement of cognitive function through food/supplements is gaining popularity worldwide; smart drug nootropic drug (nootropic drug or cognitive enhancer) is the most popular modality. Recently, there was a research paper that elaborated on the hypoperfusion of the hippocampus induced by glycemic variability [1]. It has been reported that the antioxidative supplement is also effective in improving memory [2]. Moreover, smart drugs mainly claim to “enhance brain function” and have neuropsychiatric effects [3]. Currently, they are considered as health supplements; however, their role is not yet clear. The use of smart drugs has increased worldwide in recent years, and they are used extensively for improving work efficiency, concentration, and learning memory in the United States and Europe. Additionally, a survey conducted in Japan reported that approximately 30% of Japanese pharmacy students expressed positive opinions regarding artificial improvement of their abilities through the use of smart drugs [4].

The piracetam analog, aniracetam, has recently received attention due to its potential for cognitive enhancement associated with minimal reported adverse effects [5]. Both aniracetam and piracetam are prescribed drugs for dementia, Alzheimer’s disease, and other brain traumas. Nootropic drugs including piracetam and aniracetam are known to enhance synaptic plasticity or the functionality of the neurotransmitter acetylcholine. Since it strengthens blood flow to the brain, piracetam acts on multiple areas improving cognitive abilities such as brain metabolism, learning, memory, and concentration. During the 1970s, a few small, poorly designed studies suggested that piracetam may improve memory in healthy adults, but these findings have not been replicated [6,7,8]. However, it has been shown to improve memory in people with age-related mental decline but does not seem to have much benefit in healthy adults [9,10]. There are also reports that aniracetam or piracetam was administered to normal experimental animals, and the learning experiment was conducted on cognitive function [11,12,13]. Aniracetam has been shown to enhance excitatory post-synaptic potentials [14], increase excitatory post-synaptic current (EPSC) decay time [15], and augment long-term potentiation in the hippocampus [16]. On the other hand, it has been reported that the administration of piracetam significantly enhances memory learning not only in memory-impaired animals, but also in normal animals [17,18]. In addition to being involved in the cholinergic system, these drugs are reported to act as an allosteric modulator of α-amino-3-hydroxyl-5-methyl-4-isoxazole-propionate (AMPA) receptors [19]. Their efficacy in healthy individuals and the side effects and dependence, when taken for a long period of time, have not been clear. Clarifying the efficacy and harmful effects of smart drugs on healthy individuals is important from a scientific and physiological point of view.

The memory learning function must focus on the hippocampus, which is part of the limbic system called the hippocampal formation [20]. The basic mechanism of memory formation in the hippocampus is as follows: information from sensory organs such as the eyes and ears first converges on the para-hippocampal olfactory infield cortex and reaches the dentate gyrus of the hippocampal formation [21]. Information is transmitted in the order of CA3 → CA2 → CA1 and is sorted into temporarily retained form, long-term memory, or extinguished information [22]. Long-term memory information is sent to the hippocampal formation and projected onto the neocortical association area through the entorhinal cortex, followed by the formation and strengthening of the neural circuit of the cerebral cortex, thereby preserving long-term memory.

Over the years, several studies have been conducted on the molecular mechanism of synaptic plasticity, which is a basic neural function for the development of learning and memory. The neurotransmitter at the synapse of the hippocampal major pathway is glutamic acid by AMPA receptors [23]. On the contrary, acetylcholine is also involved in the memory function [24]. In a study examining the relationship between acetylcholine receptors and learning, rats treated with the muscarinic acetylcholine receptor inhibitor scopolamine to CA1 in the hippocampus had unsatisfactory scopolamine concentration-dependent inhibitory avoidance task performance [25]. Muscarinic receptors are also considered to be involved in memory learning because the inhibitor also causes amnesia in humans. Studies measuring acetylcholine secretion in the hippocampal CA1 region before and after the learning process revealed that acetylcholine levels increased during learning and remained high after learning [26].

Therefore, we tried to clarify the mechanism that affects cognitive function, assuming that it would take us closer to the actual state of memory formation. In this study, we used normal young mice to focus on the role of acetylcholine, especially the metabolic process of acetylcholine and muscarinic acetylcholine receptors, in the hippocampus, which controls memory learning, and investigated the long-term effects of smart drugs on neural circuits.

## 2. Materials and Methods

### 2.1. Animals

We analyzed the ICR strain of male mice at seven weeks of age (Charles River, Yokohama, Japan). These experiments were performed in accordance with the National Institutes of Health Guide for the Care and Use of Laboratory Animals. The Laboratory Animal Ethics Committee of Meiji Pharmaceutical University approved these experiments (No. 2704, 1 April 2017) and all efforts were made to minimize animal suffering and reduce the number of animals used in the study.

### 2.2. Drug Administration

In this study, the dosage of drugs was calculated according to each instruction in the product document. At seven weeks of age, each drug was orally administered to nine male ICR strain mice for seven weeks. The administered drugs were as follows: (i) piracetam (1.2 mg/kg bodyweight/day, UCB Pharma, A. S., Atasehir, Republic of Turkey), (ii) aniracetam (0.75 mg/kg bodyweight/day, IASIS Pharma, Kamatero, Hellenic Republic), (iii) piracetam (1.2 mg/kg bodyweight/day) and alpha-glyceryl phosphoryl choline (α-GPC; 0.3 mg/kg bodyweight/day, Swanson Health Products, Fargo, ND, USA), and (iv) aniracetam (0.75 mg/kg bodyweight/day) and α-GPC (0.3 mg/kg bodyweight/day). Saline was administered to another nine age-matched male mice, which were controls.

### 2.3. Tissue Preparation

Tissue preparation for histochemical analysis was performed according to the protocol described previously [27,28,29,30]. All animals were deeply anesthetized with isoflurane and carbon dioxide. For the histochemical and immunohistochemical analyses, three animals from each group were perfused with a fixative containing 4% paraformaldehyde in 0.1 M phosphate buffer (PB, pH 7.4). The brain samples were then removed and postfixed in the same fixative overnight at 4 °C. 

For the immuno-electron microscopic analyses, three animals from each group were perfused with a fixative containing 4% paraformaldehyde and 0.05% glutaraldehyde in 0.1 M PB (pH 7.4). The brain samples were immediately removed and postfixed in the same fixative for 24 h at 4 °C.

### 2.4. Histological and Immunohistochemical Analysis

Histological and immunohistochemical analyses were performed in accordance with the methods described previously [27,31,32,33]. For analysis using HE staining after sectioning for 4 mm thickness using Brain Slicer, the brain sections including the hippocampus were immersed in graded concentrations of ethanol, cleared in Lemosol A (Wako Pure Chemical Industries, Ltd., Osaka, Japan), and embedded in paraffin. The paraffin-embedded brain blocks were cut into 5 μm thick sections on a sliding microtome (REM-710, Yamato Kohki Industrial, Saitama, Japan). All sections were mounted on glass slides, deparaffinized with Lemosol A, and immersed in graded concentrations of ethanol and distilled water. Sections were stained with HE solutions. After staining, the sections were dehydrated with graded concentrations of ethanol, cleared with Lemosol A, and cover-slipped.

For analysis using immunohistochemical staining, brains were cut to 4 mm thickness using Brain Slicer. Brain blocks were immersed in 30% sucrose in PB. Sections were cut into 50 μm thick sections using a freezing microtome. Sections were washed in phosphate-buffered solution (PBS) containing Triton-X 100 and incubated with blocking solution containing 1% Block Ace for 60 min. Sections were incubated with each primary antibody at 4 °C for three days. Primary antibodies used in this study were as follows: (i) goat anti-choline acetyltransferase antibody (1:1000, AB144P, Millipore, Burlington, MA, USA), (ii) rabbit-M1 receptor antibody (1:1000, YCU-PS-M1, Cosmobio, Tokyo, Japan), (iii) rabbit-M3 receptor antibody (1:1000, YCU-PS-M3, Cosmobio, Japan), and (iv) rabbit-M4 receptor antibody (1:1000, YCU-PS-M4, Cosmobio, Japan). After washing, the sections were incubated in biotin-conjugated anti-goat or rabbit antibody (1:300, Vector Laboratories, Inc., Burlingame, CA, USA) for 90 min. After washing several times, sections were incubated in avidin–biotin complex solution (VECTASTAIN Elite ABC Kit, Vector Laboratories, Inc., Burlingame, CA, USA), and reacted with 3,3′-diaminobenzidine (DAB) solution. Sections were dehydrated with graded concentrations of ethanol, cleared with Lemosol A, and were cover-slipped.

For the detection of acetylcholinesterase, we used acetylcholinesterase rapid staining kit (MBL lab. Co., Ltd., Nagano, Japan). According to this protocol, we stained the acetylcholinesterase in each section. Sections were rinsed with tap water for 10 s and incubated with staining solutions at 37 °C for 5 min, followed by washing under tap water, dehydration, and mounting.

HE- and immune-stained section images were captured using a charge-coupled device (CCD) camera (BZ-X700, Keyence, Osaka, Japan).

### 2.5. Immunoelectron Microscopic Analysis

Immunoelectron microscopic analysis was performed in accordance with the methods described previously [27,31,34,35]. The brain of each mouse was cut into 50 μm thick sections using a micro slicer. Sections including the hippocampus were washed in PBS containing Triton-X 100 and incubated with blocking solution containing 1% Block Ace for 60 min. Sections were incubated with each primary antibody at 4 °C for three days. The primary antibodies used in this study were as follows: (i) goat anti-choline acetyltransferase antibody (1:1000 dilution in PBS, AB144P, Millipore) and (ii) rabbit-M1 receptor antibody (1:1000, YCU-PS-M1, Cosmobio, Japan). After washing, the sections were separated into two groups: DAB reaction group and silver-enhancement group. For DAB reaction, half of the number of sections were incubated in biotin-conjugated anti-goat or rabbit antibody (1:300, Vector Laboratories, Inc., Burlingame, CA, USA) for 90 min. After washing several times, the sections were incubated in avidin–biotin complex solution (VECTASTAIN Elite ABC Kit, Vector Laboratories, Inc., Burlingame, CA, USA), and reacted with DAB solution. For gold-colloid reaction, half of the number of sections were incubated in 5 nm gold-conjugated anti-goat antibody (1:200 dilution in PBS, BBI solutions, Cardiff, UK) for 90 min. After washing several times, all sections were immersed in osmium tetroxide (OsO_4_; TAAB Laboratories, Ltd., Aldermaston, UK) for 1 h and were dehydrated with ethanol and embedded in Epon-812 resin (TAAB Laboratories, Ltd., Aldermaston, UK). The hippocampus sections embedded in Epon-812 resin were trimmed under light microscopy. Ultrathin sections (70 nm thick) were cut with a Leica EM UC6 ultramicrotome (Leica Microsystems, Wetzlar, Germany) and were picked up on grids (Veco, Eerbeek, The Netherlands). To eliminate false positives, the non-electron-stained ultrathin sections were examined by transmission electron microscopy (JEM-1011, JEOL, Ltd., Tokyo, Japan), and the images were captured through a CCD camera.

### 2.6. Western Blot Analysis

This study was designed to examine the hippocampus of mice following smart drug administration; all proteins were prepared for Western blotting, as described previously [27,30,31]. Briefly, all animals (three animals/group) were deeply anesthetized with isoflurane and carbon dioxide, followed by perfusion of each of the four mice in each group through their left ventricle with ice-cold saline. Subsequently, their hippocampus was rapidly removed, and homogenized.

Equal concentrations of protein from each group were then subjected to sodium dodecyl sulfate–polyacrylamide gel electrophoresis and transferred onto a polyvinylidene difluoride membrane (Immobilon^TM^-P, EMD Millipore, Billerica, MA, USA). These membranes were blocked using 10% (w/v) skim milk (Becton, Dickinson and Company, Franklin Lakes, NJ, USA) in PBS containing 0.1% Tween 20 for 1 h at room temperature (RT). They were washed and incubated with a goat anti-choline acetyltransferase antibody (1:1000, AB144P, Millipore) or a rabbit anti-acetylcholinesterase antibody (1:1000, ab97299, Abcam, Cambridge, UK) for 2 h at RT. The membranes were washed and incubated with a horseradish peroxidase-conjugated anti-goat or rabbit antibody (1:2000, LI-COR Corporate, Lincoln, NE, USA) for 45 min at RT. Immunoreactive bands were detected using Western PREMIUM chemiluminescent substrate (LI-COR Corporate, NE, USA) and a LI-COR C-DiGit chemiluminescence Western blot scanner (LI-COR Corporate, NE, USA). We also used a primary antibody against α-tubulin (Sigma Aldrich, St. Louis, MO, USA) as an internal loading control.

### 2.7. Data Analysis and Measurement of Perivascular Edema

Western blot images were analyzed using ImageJ software (NIH, Bethesda, MD, USA). The densities of the immunoreactivity bands were quantified, and statistical analysis was performed using StatView statistical software (SAS Institute Inc., Cary, NC, USA). Differences were analyzed using analysis of variance (ANOVA), and statistical significance was set at *p* < 0.05.

For the measurement of perivascular edemas, total independent 40 blood vessels from both left and right sides of the hippocampus were examined per mouse (three animals/group). The criteria for the classification of the degree are 0: no perivascular edema; 1: limited perivascular edema (width of blood vessel > perivascular space); and 2: great perivascular edema (width of blood vessel < perivascular space). Differences were analyzed using ANOVA, and statistical significance was set at *p* < 0.05.

## 3. Results

### 3.1. Pathological Analysis

**Figure 1:**
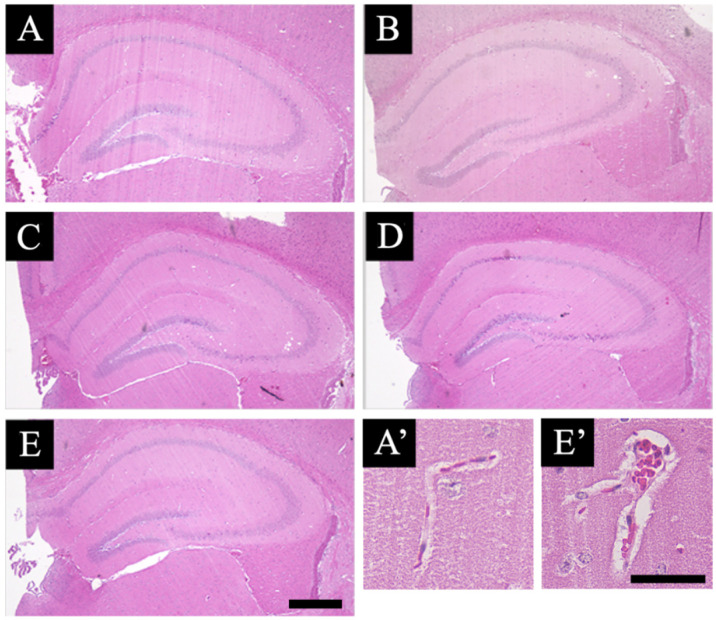
Hematoxylin and eosin (HE)-stained sections are shown: (**A**–**E**) indicate the HE-stained hippocampal sections of the vehicle-treated animals (**A**), piracetam-treated animals (**B**), aniracetam-treated animals (**C**), piracetam and α-GPC-treated animals (**D**), and aniracetam and α-GPC-treated animals (**E**). (**A’**,**E’**) indicate the blood vessels in the hippocampus of vehicle-treated animals (**A’**) and aniracetam and α-GPC-treated animals (**E’**). Scale bars in (**E**,**E’**) = 500 μm and 50 μm, respectively.

In this study, we evaluated the effect of smart drugs on the hippocampal morphology. Using hematoxylin and eosin (HE) stain, we checked the morphological changes in the hippocampus (Figure 1). There were no significant changes in any of the neurons belonging to the hippocampus sections. Compared with the investigative findings in normal specimens (Figure 1A), no findings such as increased or decreased cell number, disordered arrangement, or atrophy were seen in the drug-administered specimens (Figure 1B–E). There were also no significant changes in the pattern of HE staining.

**Figure 2:**
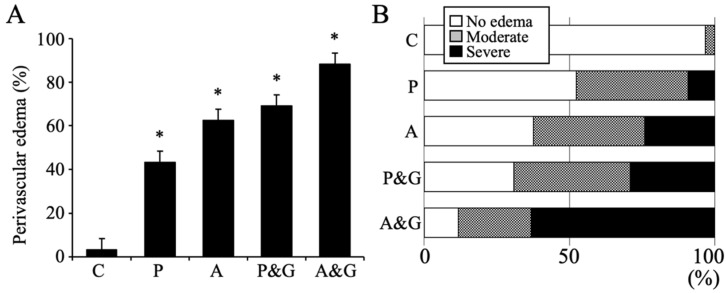
(**A**) shows the percentage of the perivascular edema in hippocampus (total of 40 blood vessels from both sides of hippocampus of each animal, 3 animals/group). Data are expressed as mean ± standard deviation. C, P, A, P&G, and A&G represent the vehicle-treated control animals, piracetam-treated animals, aniracetam-treated animals, piracetam and α-GPC-treated animals, and aniracetam and α-GPC-treated animals, respectively. *: *p* < 0.05 compared with the control. (**B**) shows the percentage of the degree of perivascular edema in hippocampus (total 40 blood vessels of each animal, 3 animals/group). The criteria for classification of the degree are white bars: no perivascular edema, gray bars: moderate perivascular edema (width of blood vessel > perivascular space), and black bars: severe perivascular edema (width of blood vessel < perivascular space).

On the contrary, perivascular swelling (edema) was observed in the blood vessel morphology of the hippocampus in all the drug-administered specimens (Figure 2A). This feature was particularly noticeable in both the aniracetam and α-GPC administration groups (control: A’, and aniracetam and α-GPC administration group: E’ in Figure 1). Moreover, the degree of perivascular edema of aniracetam and α-GPC administration animals was indicated as the most severe (Figure 2B).

### 3.2. Distribution Changes in Acetylcholinesterase

**Figure 3:**
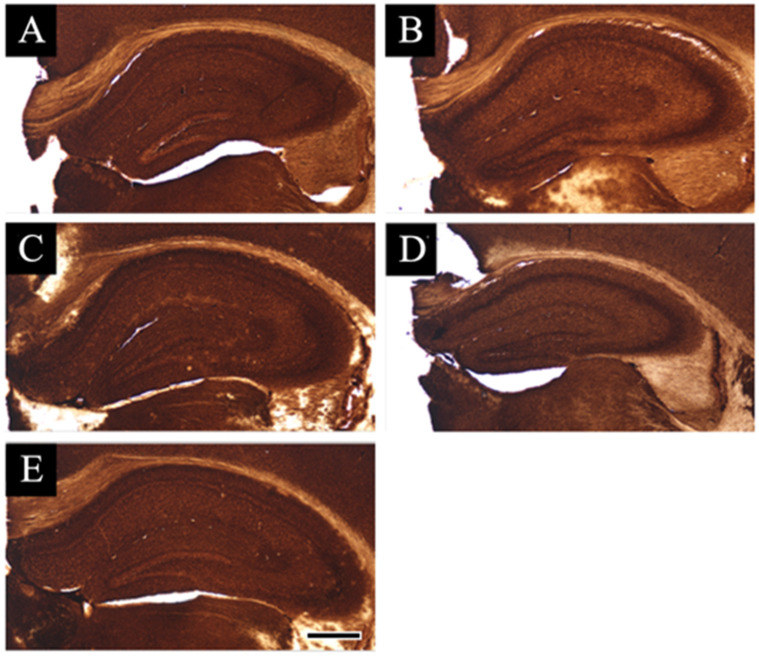
Acetylcholinesterase staining sections are shown: (**A**–**E**) indicate the acetylcholinesterase-stained hippocampal sections of the vehicle-treated animals (**A**), piracetam-treated animals (**B**), aniracetam-treated animals (**C**), piracetam and α-GPC-treated animals (**D**), aniracetam and α-GPC-treated animals (**E**). Scale bars in (**E**) = 500 μm.

We evaluated whether the staining changes in acetylcholinesterase hydrolyze the neurotransmitter acetylcholine at the cholinergic synapses in the hippocampus after each smart drug administration (Figure 3). Moreover, the hippocampus was stained well all over by acetylcholinesterase in normal animals (Figure 3A), with almost similar staining as observed in the hippocampus of other animals (Figure 3B–E). These results revealed that there were no significant changes in the acetylcholinesterase staining between the hippocampus of the normal and drug-administered mice.

### 3.3. Distribution Changes in Choline Acetyltransferase (Immunohistochemical and Immuno-Ultrastructural Analysis)

**Figure 4:**
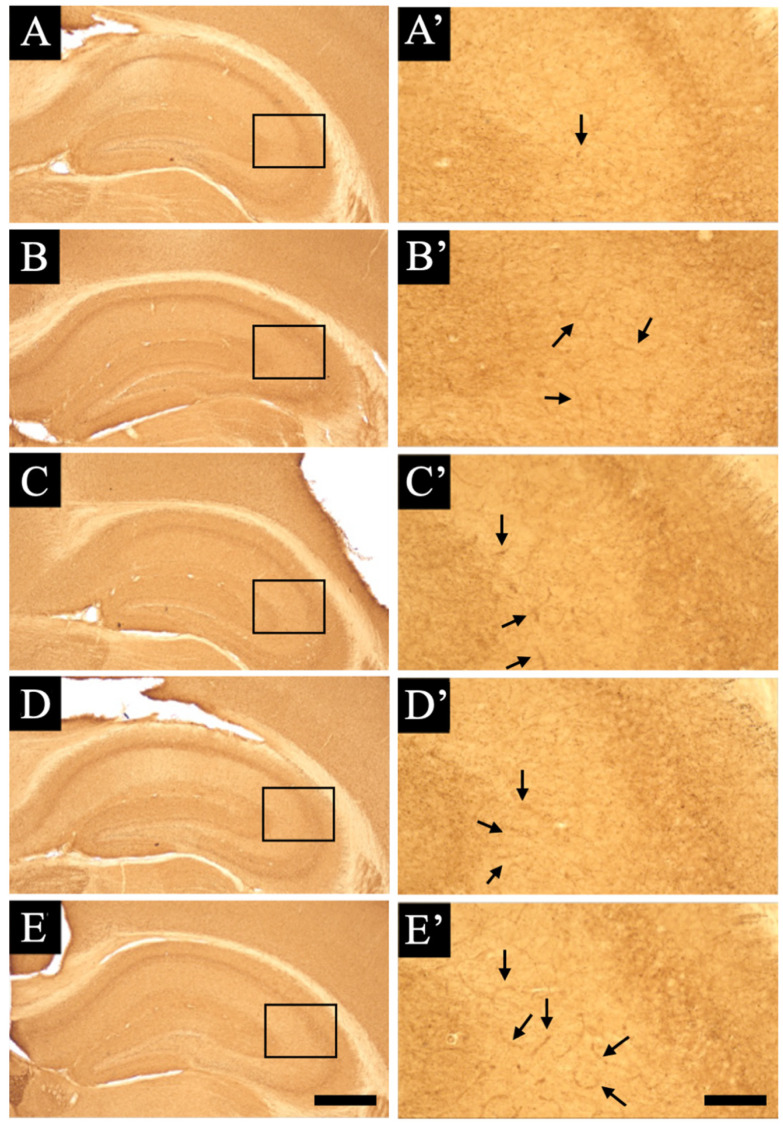
Choline acetyltransferase staining sections are shown: (**A**–**E**) are the lower magnification views, and (**A’**–**E’**) are the higher magnification views. (**A**–**E**) indicate the choline acetyltransferase-stained hippocampal sections of the vehicle-treated animals (**A**,**A’**), piracetam-treated animals (**B**,**B’**), aniracetam-treated animals (**C**,**C’**), piracetam and α-GPC-treated animals (**D**,**D’**), aniracetam and α-GPC-treated animals (**E**,**E’**), respectively. Arrows indicate the choline acetyltransferase immuno-positive staining of blood vessels. Scale bars in (**E**,**E’**) = 500 μm and 100 μm, respectively.

We demonstrated whether the expression pattern changes in choline acetyltransferase help in synthesizing the neurotransmitter acetylcholine in the hippocampus and are affected by the administration of several types of smart drugs (Figure 4). In normal animals, we found that the staining of choline acetyltransferase appeared fibrous all over the hippocampus and was relatively abundant around the neuronal cell body (Figure 4A,A’). We found that a small amount of choline acetyltransferase was expressed in or around blood vessels (arrow shown in Figure 4A’). The expression patterns of choline acetylcholinesterase with the fibrous staining were observed to be almost similar in all drug-administered animals. However, the expression of choline acetylcholinesterase in or around blood vessels was found to be increased, especially in the aniracetam and α-GPC-administered animals (arrows shown in Figure 4B–E’).

**Figure 5:**
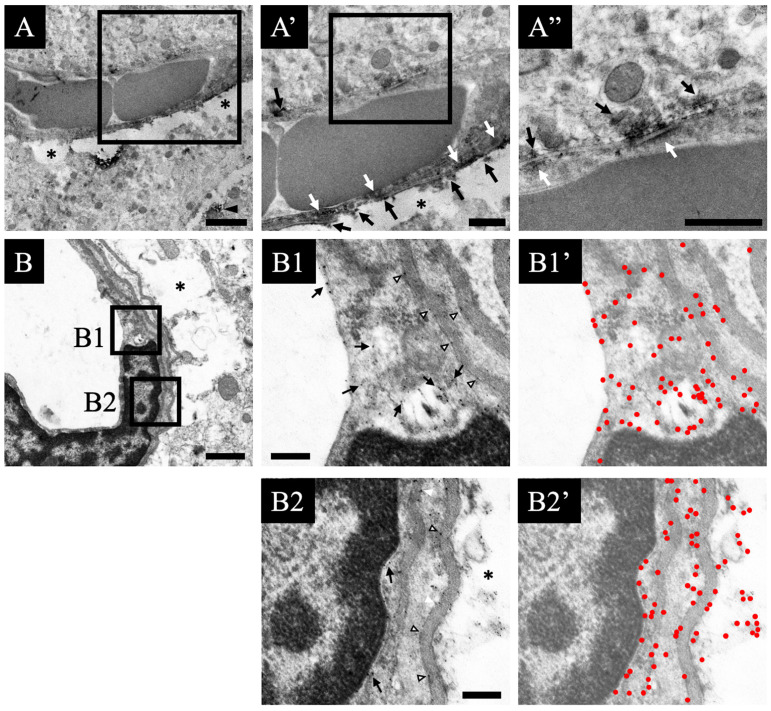
Immunoelectron microscopic images of the choline acetyltransferase staining sections of the aniracetam and α-GPC-treated animals are shown: (**A**,**B**) show the lower magnification views, (**A’**) shows the middle magnification views, and (**A”**,**B1**,**B2**) show the higher magnification views. (**A**–**A”**) indicate the DAB-reacted hippocampal sections of the aniracetam and α-GPC-treated animals. Black arrowhead in (**A**) indicates the immuno-positive sites of the cholinergic axon. Black arrows in (**A’**,**A”**) indicate the immuno-positive sites outside the basal membrane. White arrows in (**A’**,**A”**) indicate the immuno-positive sites inside the basal membrane (blood vessel side). (**B**,**B1**,**B1’**,**B2**,**B2’**) show the gold particles in the hippocampal sections of the aniracetam and α-GPC-treated animals. Black arrows in (**B1**,**B2**) indicate the immuno-positive sites of blood vessels. White arrowheads in (**B1**,**B2**) indicate the immuno-positive sites on/near the basal membrane. In (**B1’**,**B2’**), the gold particles show the red circles. Black asterisks in Figure 4 show the swellings around the blood vessels. Scale bars in (**A**,**A’**,**A”**,**B**,**B1**,**B2**) = 2 μm, 1 μm, 1 μm, 1 μm, 0.2 μm, and 0.2 μm, respectively.

We used the immuno-electron microscopic technique to detect whether the expression sites of acetylcholinesterase were in or around blood vessels in the aniracetam and α-GPC-treated animals. First, we used the DAB method, which helps observe signals similar to those of immunohistochemistry (Figure 5A–A”). The signals of choline acetyltransferase were observed in cholinergic axons (black arrowheads in Figure 5A) and blood vessels (black and white arrows in Figure 5A’,A”), both on the outside (black arrows in Figure 5A’,A”) and inside the basal membrane (white arrows in Figure 5A’,A”). To examine the signals distributed inside the basal membrane in detail, analysis was performed using the colloidal gold method (Figure 5B group). As shown in Figure 5B group, the colloidal gold particles of choline acetyltransferase were widely distributed in the vascular endothelial cells (black arrows in Figure 5B1,B2) and distributed at the basal membrane boundary (white arrowheads in Figure 5B1,B2). In addition, this ultrastructural analysis revealed swellings around the blood vessels similar to the results of histochemical analysis (perivascular space) (black asterisks in Figure 5).

### 3.4. Distribution Changes in the M1 Muscarinic Acetylcholine Receptors (Immunohistochemical and Immuno-Ultrastructural Analysis)

**Figure 6:**
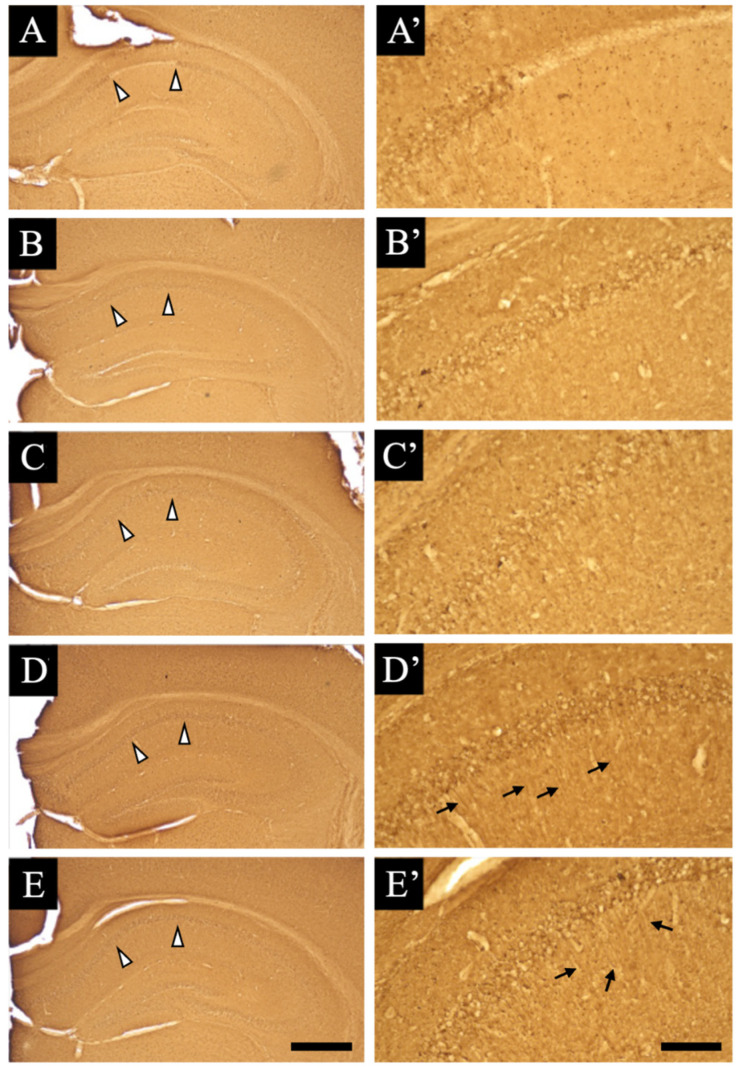
M1 muscarinic acetylcholine receptors staining sections are shown: (**A**–**E**) show the lower magnification views, (**A’**–**E’**) show the higher magnification views. (**A**–**E**) indicate the M1 muscarinic acetylcholine receptor-stained hippocampal sections of the vehicle-treated animals (**A**,**A’**), piracetam-treated animals (**B**,**B’**), aniracetam-treated animals (**C**,**C’**), piracetam and α-GPC-treated animals (**D**,**D’**), and aniracetam and α-GPC-treated animals (**E**,**E’**). White arrowheads in (**A**–**E**) indicate the CA1 region. Black arrows in (**D’**,**E’**) show the M1 muscarinic acetylcholine receptors around the dendrites and/or axons of CA1 neurons. Scale bars in (**E**,**E’**) = 500 μm and 100 μm, respectively.

To determine whether there were changes in the distribution of acetylcholine receptors, we used immunohistochemical and immuno-electron microscopic analysis. In normal animals, most M1 muscarinic acetylcholine receptors were distributed around the cell body except CA1 neurons (between white arrowheads in Figure 6A). However, in drug-administered animals, in addition to a similar expression to that of normal animals, expression of the M1 muscarinic acetylcholine receptor was also observed in CA1 (between white arrowheads in Figure 6B–E). Furthermore, in animals that also received α-GPC, several expressions of M1 muscarinic acetylcholine receptors were also observed around the dendrites and/or axons of CA1 neurons (black arrows shown in Figure 6D’,E’). Moreover, we demonstrated the immunohistochemical analysis using antibodies for the M3 and M4 muscarinic acetylcholine receptors. These results revealed that significant changes were not detected after each smart drug administration (data not shown).

**Figure 7:**
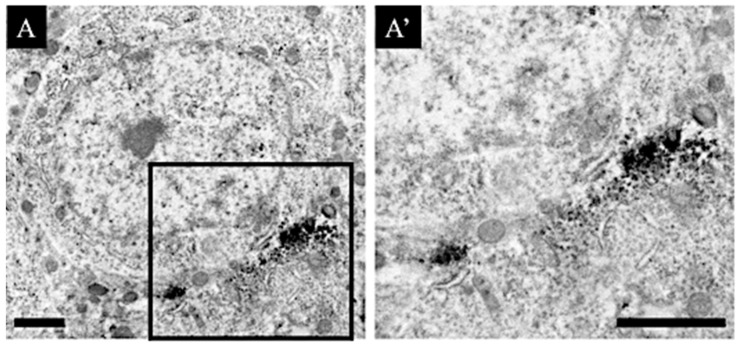
M1 muscarinic acetylcholine receptor-staining sections are shown: (**A**) shows the lower magnification view and (**A’**) shows the higher magnification view. (**A**,**A’**) indicate the DAB-reacted M1 muscarinic acetylcholine receptors of CA1 neurons of the aniracetam and α-GPC-treated animals. Scale bars in both (**A**,**A’**) = 2 μm.

We investigated whether the M1 muscarinic acetylcholine receptors that were newly expressed in the CA1 region of the aniracetam and α-GPC-treated animals are distributed using immuno-electron microscopic analysis (Figure 7). The DAB signals of M1 muscarinic acetylcholine receptors were distributed in the neuronal cell bodies in CA1. Moreover, from the shape and localization, they are assumed to be the post-synapses between cholinergic axon terminals and CA1 neurons.

### 3.5. Changes in the Expression of Acetylcholinesterase and Choline Acetyltransferase after Smart Drug Administration

**Figure 8:**
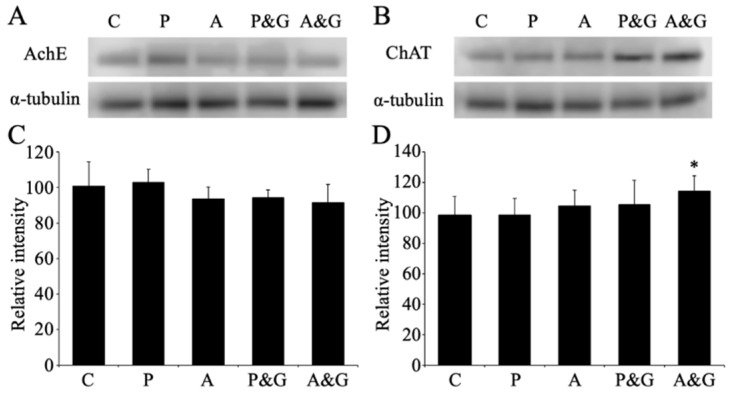
Images of Western blot of acetylcholinesterase (**A**) and choline acetyltransferase (**B**) after smart drug administration are shown. α-tubulin was used as a loading control. Densitometry analysis of the Western blot data of acetylcholinesterase and choline acetyltransferase are shown in (**C**) and (**D**), respectively. Data are expressed as mean ± standard deviation. Data are also shown as the relative intensity (100 = intensity of control value). C, P, A, P&G, and A&G represent the vehicle-treated control animals, piracetam-treated animals, aniracetam-treated animals, piracetam and α-GPC-treated animals, and aniracetam and α-GPC-treated animals, respectively. AchE: acetylcholinesterase; ChAT: choline acetyltransferase *: *p* < 0.05 compared with the control.

We evaluated the expression changes in acetylcholinesterase and choline acetyltransferase after smart drug administration using Western blot analysis (Figure 8A,B). Using densitometry analysis of the Western blot in drug-administered animals, a decreasing trend was observed in acetylcholinesterase expression; however, no significant changes were observed in acetylcholinesterase expression for each smart drug administration and control (Figure 8C). On the contrary, the expression of choline acetyltransferase after smart drug administration was significantly increased in aniracetam and α-GPC-treated animals (Figure 8D).

## 4. Discussion

In the present study, we detected the effect of long-term smart drug administration. The drug used in this experiment is a drug that has been shown to be safe and effective as a prescription drug. However, there is no report on the histopathological examination of the effect on the brain when used in healthy participants. Piracetam is a drug marketed for the treatment of myoclonus and is a cognitive enhancer [36]. Aniracetam, an analog substrate of piracetam, is four to eight times more effective than piracetam [37,38]. The functional mechanisms of piracetam and aniracetam are not completely understood. However, these drugs are known to improve the function of the neurotransmitter acetylcholine through muscarinic cholinergic receptors, which are implicated in memory processes [39]. α-GPC is also a precursor of acetylcholine and is important for the function of cholinergic neurons. Using these drugs, we evaluated the effect of long-term smart drug administration, which has been suggested to improve memory, in the hippocampal tissue.

The neurotransmitter in the hippocampal major neural circuit involved in memory learning is glutamate, and glutamate receptors are important for the induction of long-term potentiation (LTP) [40,41,42,43,44,45]. The activation of the N-methyl-D-aspartate (NMDA)-type receptor, which is a type of glutamate receptor, is involved in LTP in the CA1 region and dentate gyrus [43,44,45,46]. The activation of the muscarinic receptor revealed in this study may finally affect the activation of the NMDA receptor. In a study using rat hippocampal slice specimens [47], the change in the membrane potential of CA1 neurons associated with NMDA activation increased in a concentration-dependent manner on the administration of acetylcholine, which was not enhanced in the presence of the muscarinic receptor inhibitor atropine. Therefore, it has been suggested that muscarinic receptor activation enhances NMDA receptor activity. However, it was reported that the AMPA-type receptor is involved in memory and learning [48,49,50]. In addition, as a result of measuring the amount of acetylcholine secreted in the hippocampal CA1 region before and after learning, it was clarified that the amount of acetylcholine secreted increased during learning and remained high even after learning [26]. Moreover, this study showed that acetylcholine is involved in memory learning because inhibition of the M1 receptor also inhibits excitatory synaptic plasticity and prevents avoidance learning. Studies report that aniracetam has been shown to positively modulate the AMPA receptor [51].

**Figure 9:**
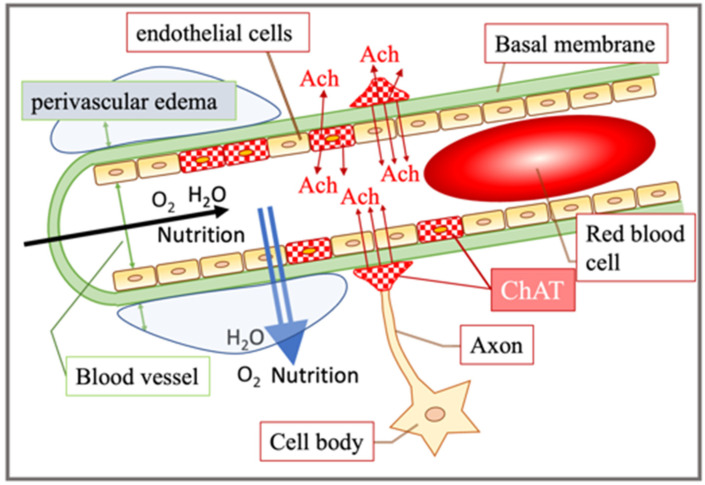
Schema of the changes in blood vessel after long-term smart drug administration is shown. Long-term smart drug administration induced ChAT expression in the vascular endothelial cells and induced perivascular edema. ChAT: choline acetyltransferase; Ach: acetylcholine.

A mechanism causing LTP of synaptic transmission involved in memory formation was reported to be enhanced by intrahippocampal acetylcholine [52]. The hippocampus receives projections of cholinergic nerves. Stimulating this cholinergic nerve to release acetylcholine increased the excitability of the hippocampal neurons. Furthermore, it is reported that when LTP is induced simultaneously, the rate of potentiation increases. Analysis using M1 receptor knockout mice also showed that the promotion of LTP by endogenous acetylcholine is mediated by the M1 receptor. This study suggests the importance of cholinergic nerves and the presence of M1 receptors in memory formation. We also created model rats in which acetylcholine was attenuated by drug administration and examined memory learning using the Morris water maze as a test for learning and memory [53]. As a result, a significant decrease in memory learning was observed in the model rats with attenuated acetylcholine in comparison to normal rats. In this model animal, in addition to diminished LTP induction in hippocampal CA1, a decrease in synaptic density was observed. These reports suggest that the formation of memory learning involves the release of acetylcholine in the hippocampus and the M1 receptor. In this study, using Western blot analysis, we clarified the expression of acetylcholinesterase and choline acetyltransferase in the mouse hippocampus after long-term administration of smart drugs (Figure 9). No significant difference was observed in acetylcholinesterase; hence, it is considered that the mechanism of decomposition of acetylcholine is normal. On the contrary, choline acetyltransferase showed a significant increase with long-term administration of smart drugs, especially in aniracetam and α-GPC-treated animals. Therefore, it was suggested that the hippocampal acetylcholine concentration increased due to the long-term administration of smart drugs. Furthermore, it was possible that activation of muscarinic receptors at least, especially M1 receptors (Figure 6), may lead to further enhancement of glutamate receptor activity, leading to LTP of synaptic transmission. It has been reported that the nicotinic acetylcholine receptor is also involved in LTP [54,55,56]. Therefore, it will be necessary to analyze the nicotinic acetylcholine receptor associated with smart drug intake.

We also found that smart drug administration caused the induction of choline acetyltransferase and mediated intracellular synthesis of acetylcholine in vascular endothelial cells (Figure 4 and Figure 5). Further studies [57,58] have shown that when acetylcholine stimulates muscarinic receptors in vascular endothelial cells, nitric oxide is produced and vasodilation occurs. As blood contains a large amount of acetylcholinesterase, the origin of acetylcholine that acts on vascular endothelial cells was previously unknown in detail. However, it was reported that vascular endothelial cells synthesize and release acetylcholine, which is subsequently transmitted [57,59]. It has been suggested that it acts not only as a substance, but also as an autacoid. Kirkpatrick et al. [60,61] found the expression of choline acetyltransferase activity and acetylcholine in human umbilical vein vascular endothelial cells. In addition, they discovered the intercellular adhesion molecule expression-promoting effect of non-nervous acetylcholine. This non-neuronal cholinergic system has been reported from bacteria to mammals, and its function is drawing attention [62,63,64,65,66,67]. In mammals, the non-neuronal acetylcholine expression has been observed in reproductive organs, immune system cells, epidermal keratinocytes, airway epithelial cells, and vascular endothelial cells [68]. Although non-neuronal acetylcholine is predicted to function as a local cell signaling or trophic molecule [69], the physiological significance of the synthesis and release of non-neuronal acetylcholine in the endothelial cells remains unclear. In intestinal epithelial cells, acetylcholine is critical in controlling the intestinal epithelial ion transport, which strongly influences water movement and hydration [70]. More recently, the non-neuronal release of acetylcholine from colonocytes coupled with propionate stimulation has been shown to play a key role in chloride secretion [71]. Therefore, in vascular endothelial cells, it is possible that acetylcholine from endothelial cells is related to the transport functions of water movement and/or chloride secretion (Figure 2 and Figure 9).

**Figure 10:**
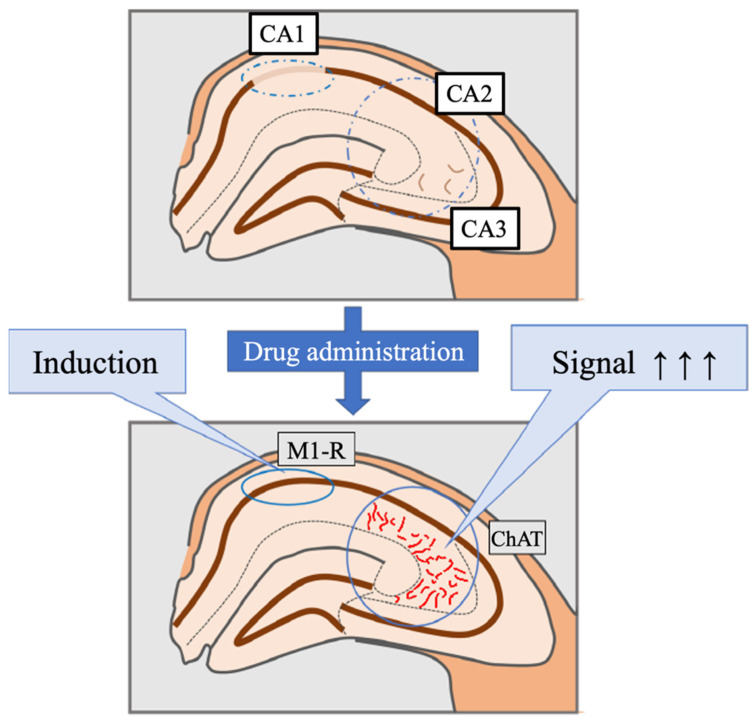
Schema of the hippocampus after long-term smart drug administration, which induced M1 receptors in CA1 and ChAT expression in the vascular endothelial cells in CA2 and CA3, are shown. M1-R: M1 muscarinic acetylcholine receptor; ChAT: choline acetyltransferase.

This study is a histological analysis of the effects of smart drug administration (Figure 10). The results revealed in this study indicate that the long-term use of smart drugs may cause dilation of blood vessels in the hippocampus and increase the influx of nutrients and oxygen to promote nerve cell activation. Moreover, M1 receptors in the CA1 neurons were newly induced. It is suggested that the LTP of synaptic transmission through muscarinic receptors to cells is induced and expressed, and there is a possibility of a new neural circuit activation mechanism mediated by acetylcholine. Based on these results, by clarifying whether these histological changes are similarly caused in the human brain, the effect of improving cognitive function by smart drugs and the mechanism of the memory-improving effect will be clarified in future studies.

## Data Availability

The datasets used and/or analyzed during the current study are available through the corresponding author upon reasonable request.

## References

[1] Alster P., Dunalska A., Migda B., Madetko N., Krolicki L. (2021). The Rate of Decrease in Brain Perfusion in Progressive Supranuclear Palsy and Corticobasal Syndrome May be Impacted by Glycemic Variability—A Pilot Study. Front. Neurol..

[2] Tadokoro K., Morihara R., Ohta Y., Hishikawa N., Kawano S., Sasaki R., Matsumoto N., Nomura E., Nakano Y., Takahashi Y. (2019). Clinical Benefits of Antioxidative Supplement Twendee X for Mild Cognitive Impairment: A Multicenter, Randomized, Double-Blind, and Placebo-Controlled Prospective Interventional Study. J. Alzheimer’s Dis..

[3] Sharif S., Guirguis A., Fergus S., Schifano F. (2021). The Use and Impact of Cognitive Enhancers among University Students: A Systematic Review. Brain Sci..

[4] Yamamoto M., Ishii Y. (2018). Awareness survey of smart drugs amoung undergraduates. Jpn. J. Drug Inform..

[5] Gouliaev A.H., Senning A. (1994). Piracetam and other structurally related nootropics. Brain Res. Rev..

[6] Wilsher C., Atkins G., Manfield P. (1979). Piracetam as an aid to learning in dyslexia. Psychopharmacology.

[7] Mindus P., Cronholm B., Levander S.E., Schalling D. (1976). Piracetam-induced improvement of mental performance. A controlled study on normally aging individuals. Acta Psychiatr. Scand..

[8] Dimond S.J., Brouwers E.M. (1976). Increase in the power of human memory in normal man through the use of drugs. Psychopharmacology.

[9] Leuner K., Kurz C., Guidetti G., Orgogozo J.M., Muller W.E. (2010). Improved mitochondrial function in brain aging and Alzheimer disease—The new mechanism of action of the old metabolic enhancer piracetam. Front. Neurosci..

[10] Waegemans T., Wilsher C.R., Danniau A., Ferris S.H., Kurz A., Winblad B. (2002). Clinical efficacy of piracetam in cognitive impairment: A meta-analysis. Dement. Geriatr. Cogn. Disord..

[11] Reynolds C.D., Jefferson T.S., Volquardsen M., Pandian A., Smith G.D., Holley A.J., Lugo J.N. (2017). Oral aniracetam treatment in C57BL/6J mice without pre-existing cognitive dysfunction reveals no changes in learning, memory, anxiety or stereotypy. F1000Research.

[12] Elston T.W., Pandian A., Smith G.D., Holley A.J., Gao N., Lugo J.N. (2014). Aniracetam does not alter cognitive and affective behavior in adult C57BL/6J mice. PLoS ONE.

[13] Phillips H., McDowell A., Mielby B.S., Tucker I.G., Colombo M. (2019). Aniracetam does not improve working memory in neurologically healthy pigeons. PLoS ONE.

[14] Isaacson J.S., Nicoll R.A. (1991). Aniracetam reduces glutamate receptor desensitization and slows the decay of fast excitatory synaptic currents in the hippocampus. Proc. Natl. Acad. Sci. USA.

[15] Kolta A., Lynch G., Ambros-Ingerson J. (1998). Effects of aniracetam after LTP induction are suggestive of interactions on the kinetics of the AMPA receptor channel. Brain Res..

[16] Satoh M., Ishihara K., Iwama T., Takagi H. (1986). Aniracetam augments, and midazolam inhibits, the long-term potentiation in guinea-pig hippocampal slices. Neurosci. Lett..

[17] Kim S.K., Ko Y.H., Lee S.Y., Jang C.G. (2020). Memory-enhancing effects of 7,3′,4′-trihydroxyisoflavone by regulation of cholinergic function and BDNF signaling pathway in mice. Food Chem. Toxicol..

[18] Said E.S., Elsayed A.M., Rashed L.A., Nadwa E.H., Alsuhaibani N.A., Alfuraih B.S., Mahmoud R.H. (2021). Evaluation of nootropic activity of telmisartan and metformin on diazepam-induced cognitive dysfunction in mice through AMPK pathway and amelioration of hippocampal morphological alterations. Eur. J. Pharmacol..

[19] Ahmed A.H., Oswald R.E. (2010). Piracetam defines a new binding site for allosteric modulators of alpha-amino-3-hydroxy-5-methyl-4-isoxazole-propionic acid (AMPA) receptors. J. Med. Chem..

[20] Scoville W.B., Milner B. (1957). Loss of recent memory after bilateral hippocampal lesions. J. Neurol. Neurosurg. Psychiatry.

[21] Andersen P., Morris R., Amaral D., Bliss T., O’Keefe J. (2006). The Hippocampus Book.

[22] Lee J.C., Park J.H., Ahn J.H., Park J., Kim I.H., Cho J.H., Shin B.N., Lee T.K., Kim H., Song M. (2018). Effects of chronic scopolamine treatment on cognitive impairment and neurofilament expression in the mouse hippocampus. Mol. Med. Rep..

[23] Meldrum B.S. (2000). Glutamate as a neurotransmitter in the brain: Review of physiology and pathology. J. Nutr..

[24] Fukatsu T., Miyake-Takagi K., Nagakura A., Omino K., Okuyama N., Ando T., Takagi N., Furuya Y., Takeo S. (2002). Effects of nefiracetam on spatial memory function and acetylcholine and GABA metabolism in microsphere-embolized rats. Eur. J. Pharmacol..

[25] Zarrindast M.R., Ardjmand A., Ahmadi S., Rezayof A. (2012). Activation of dopamine D1 receptors in the medial septum improves scopolamine-induced amnesia in the dorsal hippocampus. Behav. Brain Res..

[26] Mitsushima D., Sano A., Takahashi T. (2013). A cholinergic trigger drives learning-induced plasticity at hippocampal synapses. Nat. Commun..

[27] Nakadate K., Imamura K., Watanabe Y. (2006). Cellular and subcellular localization of alpha-1 adrenoceptors in the rat visual cortex. Neuroscience.

[28] Ehara A., Taguchi D., Nakadate K., Ueda S. (2021). Attractin deficiency causes metabolic and morphological abnormalities in slow-twitch muscle. Cell Tissue Res..

[29] Taguchi D., Ehara A., Kadowaki T., Sakakibara S.I., Nakadate K., Hirata K., Ueda S. (2020). Minocycline Alleviates Cluster Formation of Activated Microglia and Age-dependent Dopaminergic Cell Death in the Substantia Nigra of Zitter Mutant Rat. Acta Histochem. Cytochem..

[30] Nakadate K., Kamata S. (2022). Severe Acute Hepatic Dysfunction Induced by Ammonium Acetate Treatment Results in Choroid Plexus Swelling and Ventricle Enlargement in the Brain. Int. J. Mol. Sci..

[31] Nakadate K. (2015). Developmental changes in the flotillin-1 expression pattern of the rat visual cortex. Neuroscience.

[32] Nakadate K., Imamura K., Watanabe Y. (2013). c-Fos activity mapping reveals differential effects of noradrenaline and serotonin depletion on the regulation of ocular dominance plasticity in rats. Neuroscience.

[33] Sano K., Nakadate K., Hanada K. (2020). Minocycline prevents and repairs the skin disorder associated with afatinib, one of the epidermal growth factor receptor-tyrosine kinase inhibitors for non-small cell lung cancer. BMC Cancer.

[34] Nakadate K., Tanaka-Nakadate S. (2015). Three-Dimensional Electron Microscopy Reconstruction of Degenerative Dopaminergic Neurons Surrounded by Activated Microglia in Substantia Nigra. Ultrastruct. Pathol..

[35] Ishii J., Sato-Yazawa H., Kashiwagi K., Nakadate K., Iwamoto M., Kohno K., Miyata-Hiramatsu C., Masawa M., Onozaki M., Noda S. (2022). Endocrine secretory granule production is caused by a lack of REST and intragranular secretory content and accelerated by PROX1. J. Mol. Histol..

[36] Cohen P.A., Zakharevich I., Gerona R. (2020). Presence of Piracetam in Cognitive Enhancement Dietary Supplements. JAMA Intern. Med..

[37] Stancheva S.L., Petkov V.D., Hadjiivanova C.I., Petkov V.V. (1991). Age-related changes of the effects of a group of nootropic drugs on the content of rat brain biogenic monoamines. Gen. Pharmacol..

[38] Saletu B., Grunberger J. (1985). Memory dysfunction and vigilance: Neurophysiological and psychopharmacological aspects. Ann. N. Y. Acad. Sci..

[39] Winnicka K., Tomasiak M., Bielawska A. (2005). Piracetam—An old drug with novel properties?. Acta Pol. Pharm..

[40] Bliss T.V., Lomo T. (1973). Long-lasting potentiation of synaptic transmission in the dentate area of the anaesthetized rabbit following stimulation of the perforant path. J. Physiol..

[41] Bliss T.V., Gardner-Medwin A.R. (1973). Long-lasting potentiation of synaptic transmission in the dentate area of the unanaestetized rabbit following stimulation of the perforant path. J. Physiol..

[42] Sumi T., Harada K. (2020). Mechanism underlying hippocampal long-term potentiation and depression based on competition between endocytosis and exocytosis of AMPA receptors. Sci. Rep..

[43] Ge Y., Dong Z., Bagot R.C., Howland J.G., Phillips A.G., Wong T.P., Wang Y.T. (2010). Hippocampal long-term depression is required for the consolidation of spatial memory. Proc. Natl. Acad. Sci. USA.

[44] Kemp A., Manahan-Vaughan D. (2007). Hippocampal long-term depression: Master or minion in declarative memory processes?. Trends Neurosci..

[45] Whitlock J.R., Heynen A.J., Shuler M.G., Bear M.F. (2006). Learning induces long-term potentiation in the hippocampus. Science.

[46] Luscher C., Malenka R.C. (2012). NMDA receptor-dependent long-term potentiation and long-term depression (LTP/LTD). Cold Spring Harb. Perspect. Biol..

[47] Markram H., Segal M. (1990). Acetylcholine potentiates responses to N-methyl-D-aspartate in the rat hippocampus. Neurosci. Lett..

[48] Park P., Kang H., Sanderson T.M., Bortolotto Z.A., Georgiou J., Zhuo M., Kaang B.K., Collingridge G.L. (2019). On the Role of Calcium-Permeable AMPARs in Long-Term Potentiation and Synaptic Tagging in the Rodent Hippocampus. Front. Synaptic. Neurosci..

[49] Park P., Kang H., Sanderson T.M., Bortolotto Z.A., Georgiou J., Zhuo M., Kaang B.K., Collingridge G.L. (2018). The Role of Calcium-Permeable AMPARs in Long-Term Potentiation at Principal Neurons in the Rodent Hippocampus. Front. Synaptic. Neurosci..

[50] Mitsushima D., Ishihara K., Sano A., Kessels H.W., Takahashi T. (2011). Contextual learning requires synaptic AMPA receptor delivery in the hippocampus. Proc. Natl. Acad. Sci. USA.

[51] Ito I., Tanabe S., Kohda A., Sugiyama H. (1990). Allosteric potentiation of quisqualate receptors by a nootropic drug aniracetam. J. Physiol..

[52] Shinoe T., Matsui M., Taketo M.M., Manabe T. (2005). Modulation of synaptic plasticity by physiological activation of M1 muscarinic acetylcholine receptors in the mouse hippocampus. J. Neurosci..

[53] Matsukawa M., Ogawa M., Nakadate K., Maeshima T., Ichitani Y., Kawai N., Okado N. (1997). Serotonin and acetylcholine are crucial to maintain hippocampal synapses and memory acquisition in rats. Neurosci. Lett..

[54] Koukouli F., Maskos U. (2015). The multiple roles of the alpha7 nicotinic acetylcholine receptor in modulating glutamatergic systems in the normal and diseased nervous system. Biochem. Pharmacol..

[55] Drever B.D., Riedel G., Platt B. (2011). The cholinergic system and hippocampal plasticity. Behav. Brain Res..

[56] Fujii S., Ji Z., Sumikawa K. (2000). Inactivation of alpha7 ACh receptors and activation of non-alpha7 ACh receptors both contribute to long term potentiation induction in the hippocampal CA1 region. Neurosci. Lett..

[57] Kawashima K., Watanabe N., Oohata H., Fujimoto K., Suzuki T., Ishizaki Y., Morita I., Murota S. (1990). Synthesis and release of acetylcholine by cultured bovine arterial endothelial cells. Neurosci. Lett..

[58] Parnavelas J.G., Kelly W., Burnstock G. (1985). Ultrastructural localization of choline acetyltransferase in vascular endothelial cells in rat brain. Nature.

[59] Ikeda C., Morita I., Mori A., Fujimoto K., Suzuki T., Kawashima K., Murota S. (1994). Phorbol ester stimulates acetylcholine synthesis in cultured endothelial cells isolated from porcine cerebral microvessels. Brain Res..

[60] Kirkpatrick C.J., Bittinger F., Nozadze K., Wessler I. (2003). Expression and function of the non-neuronal cholinergic system in endothelial cells. Life Sci..

[61] Kirkpatrick C.J., Bittinger F., Unger R.E., Kriegsmann J., Kilbinger H., Wessler I. (2001). The non-neuronal cholinergic system in the endothelium: Evidence and possible pathobiological significance. Jpn. J. Pharmacol..

[62] Wessler I., Kilbinger H., Bittinger F., Kirkpatrick C.J. (2001). The biological role of non-neuronal acetylcholine in plants and humans. Jpn. J. Pharmacol..

[63] Kawashima K., Fujii T. (2000). Extraneuronal cholinergic system in lymphocytes. Pharmacol. Ther..

[64] Wessler I., Kirkpatrick C.J., Racke K. (1999). The cholinergic ‘pitfall’: Acetylcholine, a universal cell molecule in biological systems, including humans. Clin. Exp. Pharmacol. Physiol..

[65] Wessler I., Kirkpatrick C.J., Racke K. (1998). Non-neuronal acetylcholine, a locally acting molecule, widely distributed in biological systems: Expression and function in humans. Pharmacol. Ther..

[66] Grando S.A. (1997). Biological functions of keratinocyte cholinergic receptors. J. Investig. Dermatol. Symp. Proc..

[67] Sastry B.V., Sadavongvivad C. (1978). Cholinergic systems in non-nervous tissues. Pharmacol. Rev..

[68] Grando S.A., Kawashima K., Wessler I. (2003). Introduction: The non-neuronal cholinergic system in humans. Life Sci..

[69] Klapproth H., Reinheimer T., Metzen J., Munch M., Bittinger F., Kirkpatrick C.J., Hohle K.D., Schemann M., Racke K., Wessler I. (1997). Non-neuronal acetylcholine, a signalling molecule synthezised by surface cells of rat and man. Naunyn. Schmiedeberg’s Arch. Pharmacol..

[70] Hirota C.L., McKay D.M. (2006). Cholinergic regulation of epithelial ion transport in the mammalian intestine. Br. J. Pharmacol..

[71] Yajima T., Inoue R., Matsumoto M., Yajima M. (2011). Non-neuronal release of ACh plays a key role in secretory response to luminal propionate in rat colon. J. Physiol..

